# Integrated Identification and Immunotherapy Response Analysis of the Prognostic Signature Associated With m6A, Cuproptosis‐Related, Ferroptosis‐Related lncRNA in Endometrial Cancer

**DOI:** 10.1002/cnr2.70009

**Published:** 2024-09-26

**Authors:** Yongkang Qian, Hualing Chen, Pengcheng Miao, Rongji Ma, Beier Lu, Chenhua Hu, Ru Fan, Biyun Xu, Bingwei Chen

**Affiliations:** ^1^ Department of Epidemiology and Biostatistics, School of Public Health Southeast University Nanjing China; ^2^ Medical Statistics and Analysis Center, Nanjing Drum Tower Hospital The Affiliated Hospital of Nanjing University Medical School Nanjing China

**Keywords:** cuproptosis, lncRNA, machine learning, survival risk model, women's cancer

## Abstract

**Background:**

Endometrial cancer (EC) stands as the predominant gynecological malignancy impacting the female reproductive system on a global scale. N6‐methyladenosine, cuproptosis‐ and ferroptosis‐related biomarker is beneficial to the prognostic of tumor patients. Nevertheless, the correlation between m6A‐modified lncRNAs and ferroptosis, copper‐induced apoptosis in the initiation and progression of EC remains unexplored in existing literature.

**Aims:**

In this study, based on bioinformatics approach, we identified lncRNAs co‐expressing with cuproptosis‐, ferroptosis‐, m6A‐ related lncRNAs from expression data of EC. By constructing the prognosis model in EC, we screened hub lncRNA signatures affecting prognosis of EC patients. Furthermore, the guiding value of m6A‐modified ferroptosis‐related lncRNA (mfrlncRNA) features was assessed in terms of prognosis, immune microenvironment, and drug sensitivity.

**Method:**

Our research harnessed gene expression data coupled with clinical insights derived from The Cancer Genome Atlas (TCGA) collection. To forge prognostic models, we adopted five machine learning approaches, assessing their efficacy through C‐index and time‐independent ROC analysis. We pinpointed prognostic indicators using the LASSO Cox regression approach. Moreover, we delved into the biological and immunological implications of the discovered lncRNA prognostic signatures.

**Results:**

The survival rate for the low‐risk group was markedly higher than that for the high‐risk group, as evidenced by a significant log‐rank test (*p* < 0.001). The LASSO Cox regression model yielded concordance indices of 0.76 for the training set and 0.77 for the validation set, indicating reliable prognostic accuracy. Enrichment analysis of gene functions linked the identified signature predominantly to endopeptidase inhibitor activity, highlighting the signature's potential implications. Additionally, immune function and drug density emphasized the importance of early diagnosis in EC.

**Conclusion:**

Five hub lncRNAs in EC were identified through constructing the prognosis model. Those genes might be potential biomarkers to provide valuable reference for targeted therapy and prognostic assessment of EC.

## 1 Introduction

Endometrial cancer (EC) stands as the most common gynecological malignancy impacting the female reproductive system on a global scale [1]. While early‐stage EC patients generally exhibit favorable prognoses following treatment [2, 3], the survival rates of those in advanced, metastatic, or recurrent stages significantly decrease [3, 4]. The mortality rate of EC is growing at a faster pace than its incidence rate, resulting in an estimated 76 000 female deaths globally each year [5]. The escalation in disease‐related mortality correlates with advanced stage, aggressive histology, and the occurrence of metastasis [6]. For patients with EC who are deemed suitable candidates for surgical intervention, standard treatment recommendations include total hysterectomy and bilateral salpingo‐oophorectomy, followed by lymph node staging [7]. However, late‐stage EC patients who have already received standard management often lack effective treatment options [8]. Despite progress and collaboration among various disciplines and institutions, the development and success of prognostic biomarkers remain limited [9]. Hence, it is imperative to identify predictive and prognostic features in high‐risk patients to further enhance the overall prognosis of individuals with EC.

Long noncoding RNAs (lncRNAs), characterized by their inability to encode proteins and lengths exceeding 200 nucleotides, are integral to a myriad of cellular functions [10]. Despite not encoding proteins directly, lncRNAs are considered to have essential functions in numerous cellular processes through various mechanisms [11]. Abnormal expression of lncRNAs in many tumors can result in tumor progression, metastasis, drug resistance, and other processes [12, 13]. Evidence suggests lncRNA features can effectively predict the prognosis of EC patients and further improve their survival rates [14, 15]. N6‐methyladenosine (m6A) is a prevalent RNA modification that widely exists in lncRNA, which participates in regulating the process of RNA encoding proteins [16, 17]. It exerts a significant influence on oncogenesis, growth, and metastasis. Studies have shown that m6A methylation can function as a biological indicator for early tumor diagnosis and prognosis evaluation, contributing to the improvement of patients' survival outcomes [18, 19, 20]. Furthermore, m6A modification has the capacity to regulate tumor microenvironment penetration and immune system activation, potentially impacting the efficacy of immunotherapy.

Ferroptosis and cuproptosis are both recently discovered regulated forms of programmed cell death. Ferroptosis is a cell death pathway caused by the accumulation of cytotoxic lipid peroxides regulated by iron dependency [21, 22]. The interplay of ferroptosis in tumorigenesis and resistance to systemic therapies is underscored by the role of specific proteins; for example, ferroptosis suppressor protein 1 (FSP1), which attenuates ferroptotic cell death by modulating levels of the lipid peroxidation‐associated molecule, coenzyme Q10 (CoQ10) [23]. Additionally, among all gynecological cancers, EC is particularly associated with metabolic abnormalities such as obesity, hyperglycemia, and hypertension [24]. Studies suggest that metabolic changes during obesity may render EC more sensitive to ferroptosis inducers [25].Copper, an indispensable micronutrient, is a catalyst and structural component essential for a plethora of biochemical reactions vital to life, encompassing energy metabolism, antioxidant defenses, and mitochondrial respiration [26]. However, an overabundance of copper can precipitate adverse outcomes. In various cancer types, including EC, heightened copper concentrations have been documented [27]. The oxidative stress engendered by copper may induce angiogenesis in EC, leading to the proliferation of new vasculature from a previously dormant state, thus fueling tumor progression [28]. Excess copper ions can also interact with lipid‐acylated components within the tricarboxylic acid (TCA) cycle, culminating in protein aggregation and the disintegration of iron–sulfur cluster proteins, which initiate proteotoxic stress and cell death [29]. This interaction triggers a cascade of toxic protein stress, culminating in cellular demise. This mechanism highlights the critical role of copper homeostasis in cellular metabolism and its potential impact on pathological states [26, 29]. Notably, targeting the regulation of ferroptosis‐related proteins has become an effective therapeutic approach to induce apoptosis in tumor cells, particularly for radioresistant and drug‐resistant malignant tumors [30, 31]. Adverse effects may occur if the copper content in the body passes the certain limit that body can tolerate. Elevated copper levels were observed in various types of malignancy, including EC [27]. Therefore, ferroptosis and cuproptosis related signatures may be the potential targets for the development of promising biomarkers.

During the development of endometrial carcinoma, ferroptosis and cuproptosis may also be influenced by m6A modifications on lncRNAs. However, the association between m6A‐modified lncRNAs and ferroptosis, as well as cuproptosis, in the onset and progression of EC remains unexplored. In this study, based on bioinformatics approach, we identified lncRNAs co‐expressing with cuproptosis‐, ferroptosis‐, and m6A‐related lncRNAs from expression data of EC. By constructing the prognosis model in EC, we screened hub lncRNA signatures affecting prognosis of EC patients. Furthermore, the guiding value of m6A‐modified ferroptosis‐related lncRNA (mfrlncRNA) features was assessed in terms of prognosis, immune microenvironment, and drug sensitivity. Our finding would provide valuable reference for effective targeted therapy and prognostic assessment of EC.

## 2 Materials and Methods

### 2.1 Data Collection and Processing

**FIGURE 1:**
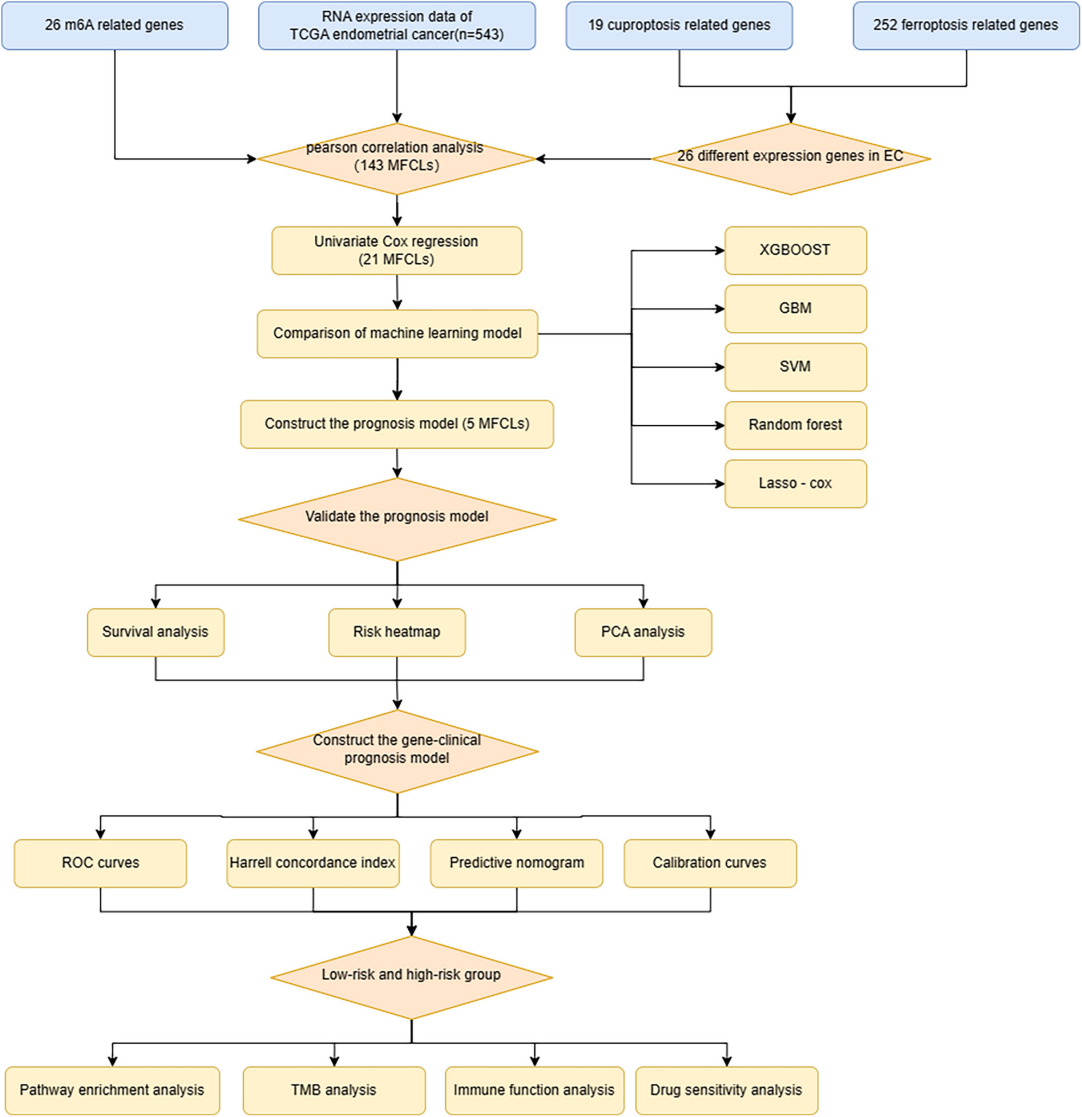
The workflow of study.

RNA‐sequencing data for 543 patients with EC was gathered from TCGA database (https://portal.gdc.cancer.gov). Clinical information of EC patients, such as age at initial pathological diagnosis, race, grade, and metastatic stage, was downloaded through the Bioconductor package “TCGAbiolinks” [32]. Five cases of missing survival status were observed and subsequently removed from the TCGA cohort. Through the “caret” package in R, our clinical dataset underwent a randomized division into training and testing sets (Figure 1).

### 2.2 Acquisition of the M6A, Ferroptosis‐ and Cuproptosis‐Related lncRNA (mfclncRNA) Pairs

From the existing literature, we identified 19 cuproptosis‐related genes (CRGs), 255 ferroptosis‐related genes (FRGs), and 26 m6A‐related genes, all selected based on a stringent false discovery rate (FDR) criterion of less than 0.05 [29]. Utilizing the “limma” package in R, we performed differential expression analysis to pinpoint 26 differentially expressed genes among the CRGs and FRGs, adhering to a defined threshold of log2 |fold change|. Subsequently, lncRNA expression profiles were correlated with these identified genes (CRGs, FRGs, and m6A‐related genes), applying a correlation coefficient threshold greater than 0.4 and a significance level of *p* < 0.05 for inclusion.

### 2.3 Construct the lncRNA Risk Model

In order to screen out lncRNAs which individually influence the prognosis of EC patients, we combined cuproptosis‐related lncRNAs with gene expression data and corresponding survival information from TCGA cohort. Subsequently, we conducted univariate Cox regression using the R package “survival.”

The expression functions of these lncRNAs sometimes are similar. To mitigate the risk of overfitting, we employed the glmnet R package to refine our analysis and adjust for complexity using the least absolute shrinkage and selection operator (LASSO) method within Cox regression analysis. This approach ensures a more robust and generalizable model by selectively shrinking coefficients to zero, thereby simplifying the model complexity [33, 34]. The lncRNAs that remained significant after this process were further evaluated using stepwise Cox regression analysis to construct a predictive risk score equation.

(1) $$\text{riskscore}=\sum_{i=1}^{p}\text{coef}\left({\text{lncRNA}}_{i}\right)\times {\text{lncRNA}}_{i}$$

In the domain of machine learning model development, this study leveraged the mlr3 R package to construct various predictive models, including the random survival forest, XGBoost, support vector machine (SVM), and generalized boosted regression model (GBM). During the model training phase, a rigorous five‐fold cross‐validation strategy was implemented on the training dataset. The hyperparameter tuning was meticulously conducted, with the aim of maximizing the concordance index (C‐index), which served as the primary performance benchmark. By employing a comprehensive grid search algorithm, the optimal hyperparameters for each model were systematically identified. Through a comparative analysis of their respective C‐indices, the most efficacious machine learning model was discerned and selected [35]. Subsequently, we will conduct a comparative analysis of these models. The more effective model is identified by comparison of these models. Following this comparative assessment, patients will be dichotomized into high‐risk and low‐risk categories according to the median risk score derived from the optimal prognostic model.

### 2.4 Validation of the mfclncRNA Prognostic Model

The Kaplan–Meier (K‐M) method was performed to estimate survival rates, which were assessed between high‐risk and low‐risk groups using the log‐rank test. To delineate the distinctions between high‐risk and low‐risk patients within the prognostic model, principal component analysis (PCA) was conducted utilizing the scatterplot3d R package. This method facilitated a three‐dimensional visualization of the patient groups' separation.

To ascertain the clinical relevance of risk scores, we combined these scores with key clinical attributes—age, histological grade, and clinical stage—within the prognostic model. This approach allowed us to develop a Cox regression model integrating gene and clinical prognostics, subsequently visualized through a forest plot in the TCGA cohort. The accuracy of our prognostic model was assessed by calculating the Harrell C‐index and constructing calibration curves. To predict overall survival (OS) at particular time intervals, we plotted a predictive nomogram utilizing the rms package. This model underwent rigorous validation against an internal testing set, ensuring its prognostic validity.

### 2.5 Gene Function Enrichment Analysis

To elucidate the biological functions and pathways of different expressed signatures between high‐risk and low‐risk groups, we performed gene enrichment analysis including the Gene Ontology (GO) and Kyoto Encyclopedia of Genes and Genomes (KEGG) pathways analysis. The GO analyses included cellular component (CC), molecular function (MF), and biological process (BP), etc. These analyses were executed using the clusterProfiler and org.Hs.eg.db R packages, facilitating sophisticated bioinformatic profiling and annotation of gene clusters [36]. FDR less than 0.05 was set as the definition of significant enrichment GO or KEGG terminology.

### 2.6 Analysis of the Tumor Mutation Burden

Tumor mutational burden (TMB) serves as a critical biomarker for assessing the mutation rate within cancer cells. We utilized the maftools package in R to handle the mutation data, enabling us to generate waterfall plots that clearly illustrate how TMB correlates with the risk categorization of patients into low‐ and high‐risk groups. Furthermore, we focused on OS as the key endpoint to explore how different TMB levels (high and low) influence the survival rates among patients with esophageal cancer (EC).

### 2.7 Evaluation of Immune Infiltration and Immunotherapy Effectiveness

To delineate the disparities in the immune microenvironment across varying risk subgroups, the ESTIMATE algorithm [37] was employed to assess the composition of immune and stromal components within the tumor immune microenvironment (TIME), encapsulating estimated scores, immune scores (IS), and stromal scores. Additionally, the distribution of immune cell populations in EC specimens was characterized using the CIBERSORT algorithm [38]. This was complemented by a comprehensive gene set enrichment analysis (GSEA), aimed at comparing the expression levels of human leukocyte antigen (HLA) genes, as well as assessing immune cell infiltration and functionality across various risk strata in bladder cancer. The interplay between key genes and immune infiltrates was further elucidated through Pearson correlation analysis, providing insights into their potential interactions. Exploring the potential benefits of immunotherapy, differential expression analysis provided insights into the expression disparities of pivotal immunotherapy targets—PD‐1, PD‐L1, and CTLA‐4—among various risk groups.

Given the burgeoning significance of immunotherapy agents such as anti‐PD1 and anti‐CTLA4 in oncology treatments, and the tumor immune dysfunction and exclusion (TIDE) algorithm's capability to forecast immunotherapy outcomes, we undertook an analysis to assess tumor cell immune escape within our prognostic models, stratifying by low‐risk and high‐risk groups based on TIDE scores [39]. The TIDE scores, retrieved from TIDE, function as prognostic indicators, with elevated scores signifying an increased probability of immune evasion. Such phenomena are indicative of a potentially less favorable response to immunotherapy, underscoring the utility of TIDE scores in predicting patient outcomes.

### 2.8 Drug Sensitivity Analysis

To predict which common chemotherapeutic drugs are most suitable for EC patients, we compared the 50% inhibiting concentration (IC50) values between high‐risk and low‐risk groups. This comparison was conducted using the Wilcoxon signed‐rank test, facilitated by the pRRophetic package in R, to identify statistically significant differences in drug sensitivity across the groups [40].

### 2.9 Statistical Analysis

This research utilized RStudio, with R version 4.1.3, for its comprehensive statistical analysis. Chi‐square tests were applied to categorical variables, and the Wilcoxon rank‐sum test was used for continuous variables. Survival outcomes were assessed with log‐rank tests and analyzed via Cox regression. The predictive model's accuracy was summarized with the C‐index and calibration curves, aimed at discriminating between different outcomes. A threshold of a two‐sided *p* < 0.05 was set for determining statistical significance, unless otherwise specified.

## 3 Result

### 3.1 Data Processing

**FIGURE 2:**
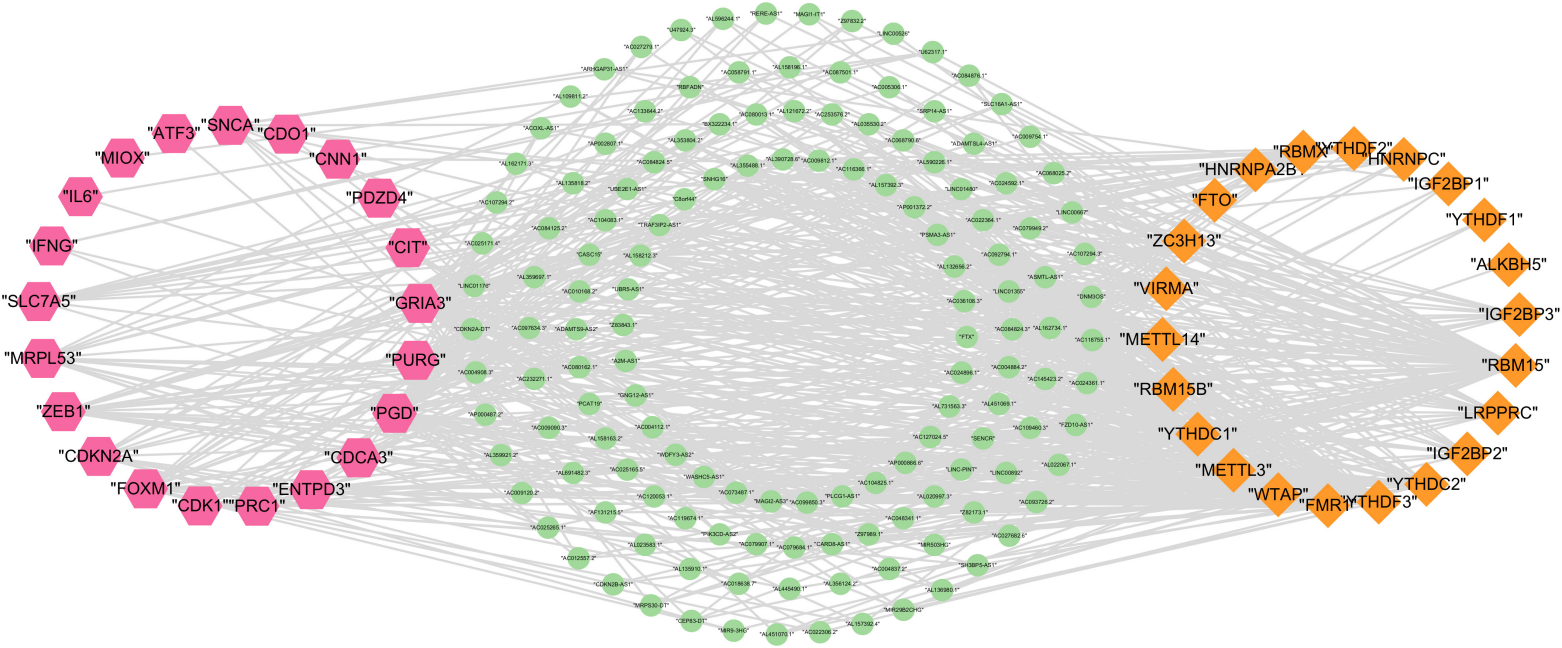
lncRNA co‐expression network.

Patients were randomly assigned to training and testing sets for our analysis. Differential expression analysis on CRGs and FRGs identified 26 ferroptosis‐ and cuproptosis‐related genes (FCRG). Co‐expression analysis further revealed 278 lncRNAs associated with FCRG and 178 lncRNAs linked to m6A methylation‐related genes. Integrating these findings, we identified 144 lncRNAs co‐expressed with MFCRG‐related genes, as detailed in Figure 2. The study involved 543 EC patients, divided into training and test sets at a ratio of 7:3, each equipped with detailed gene expression and clinical details. The staging of tumors was based on the FIGO standards, and histologic grading followed the World Health Organization's guidelines, with stages classified from I to IV and grades as G2, G3, and G4. The primary clinical attributes of the study participants are summarized in Table 1. The results demonstrate no statistical differences between training set and test set including state, stage, and grade.

**TABLE 1:** The clinical characteristics of endometrial cancer patients between training and validation group.

Variable	Overall, *N* = 543^1^	Test, *N* = 160^1^	Train, *N* = 383^1^	*p*‐value^2^
State				
Alive	452 (83%)	135 (84%)	317 (83%)	0.74
Death	91 (17%)	25 (16%)	66 (17%)	
Age				
<60	178 (33%)	52 (32%)	126 (33%)	>0.99
>60	365 (67%)	108 (68%)	257 (67%)	
BMI				
<18.5	4 (0.7%)	0 (0%)	4 (1.0%)	0.25
18.5 ~ 24	65 (12%)	14 (8.8%)	51 (13%)	
24 ~ 28	94 (17%)	28 (18%)	66 (17%)	
≥28	380 (70%)	118 (74%)	262 (68%)	
Grade				
G1	98 (18%)	33 (21%)	65 (17%)	0.60
G2	119 (22%)	34 (21%)	85 (22%)	
G3	326 (60%)	93 (58%)	233 (61%)	
G4	0 (0%)	0 (0%)	0 (0%)	
Stage				
Stage I	337 (62%)	104 (65%)	233 (61%)	0.053
Stage II	51 (9.4%)	7 (4.4%)	44 (11%)	
Stage III	126 (23%)	42 (26%)	84 (22%)	
Stage IV	29 (5.3%)	7 (4.4%)	22 (5.7%)	

^1^ *n* (%).

^2^ Pearson's chi‐squared test.

### 3.2 Identification Construction of the Prognosis Signature of mfclncRNAs

**FIGURE 3:**
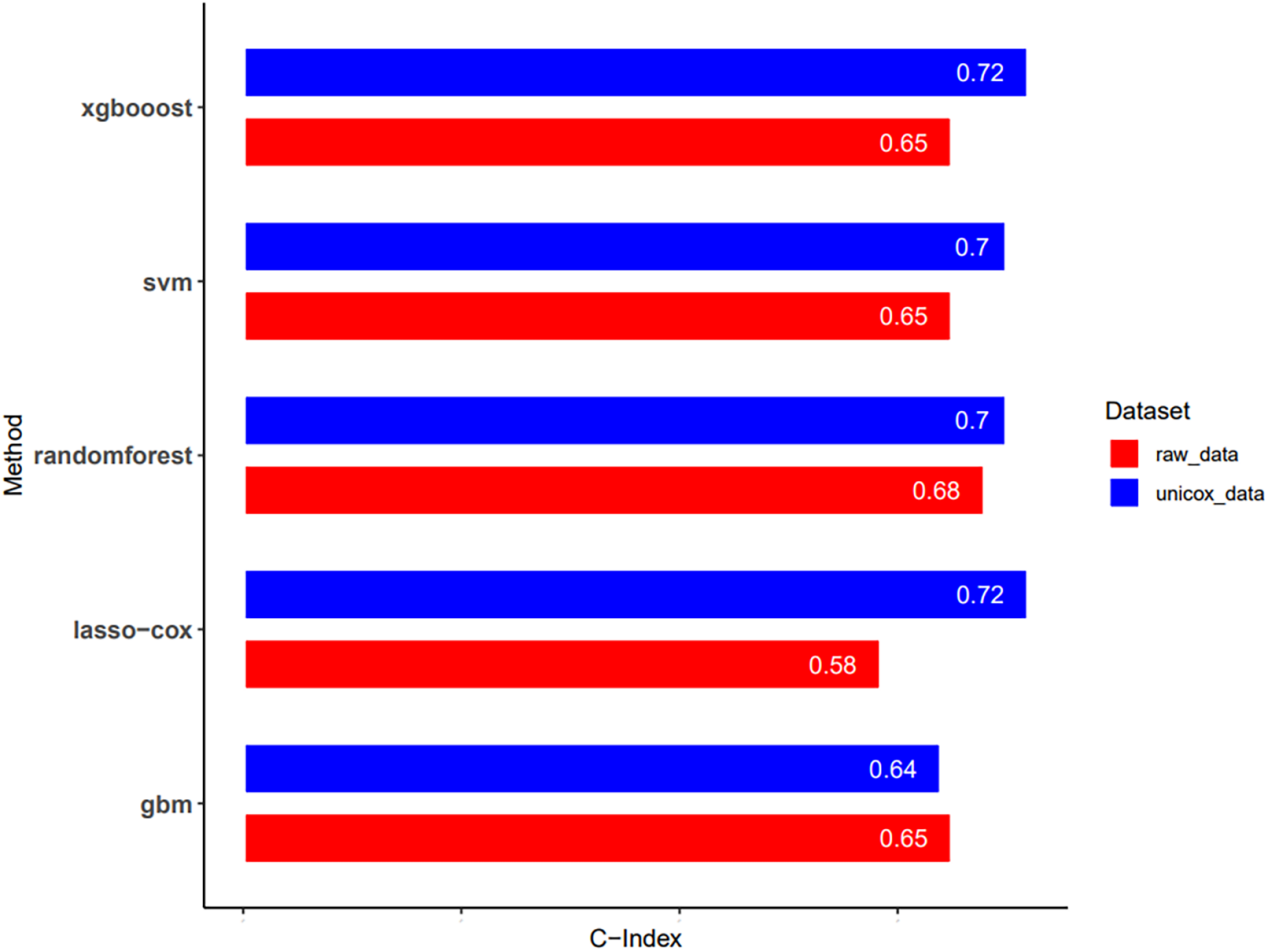
Comparison of C‐index between different model building methods.

To further explore the co‐expression lncRNAs identified, we performed univariate Cox regression and obtained 21 mfclncRNAs that significantly affected the survival status of EC patients. Subsequently, we constructed LASSO regression joint multifactor Cox regression, random survival forest, GBM, and XGBOOST models based on the mlr3 package, respectively, and determined the optimal prognostic model by calculating the corresponding C‐index in the validation set. Ultimately, the LASSO and Cox regression model had a higher C‐index, indicating it was more effective (Figure 3).

Subsequently, we identified eight lncRNAs by LASSO Cox regression. Finally, we performed stepwise Cox regression to construct the signature‐only prognosis risk model of EC patients, obtained five mfclncRNAs and corresponding risk score (Figure 2). The risk scores model was as follow: 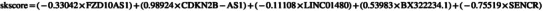. Based on the median risk scores, the patients in training set were classified into high‐risk (*n* = 192) and low‐risk groups (*n* = 192).

**FIGURE 4:**
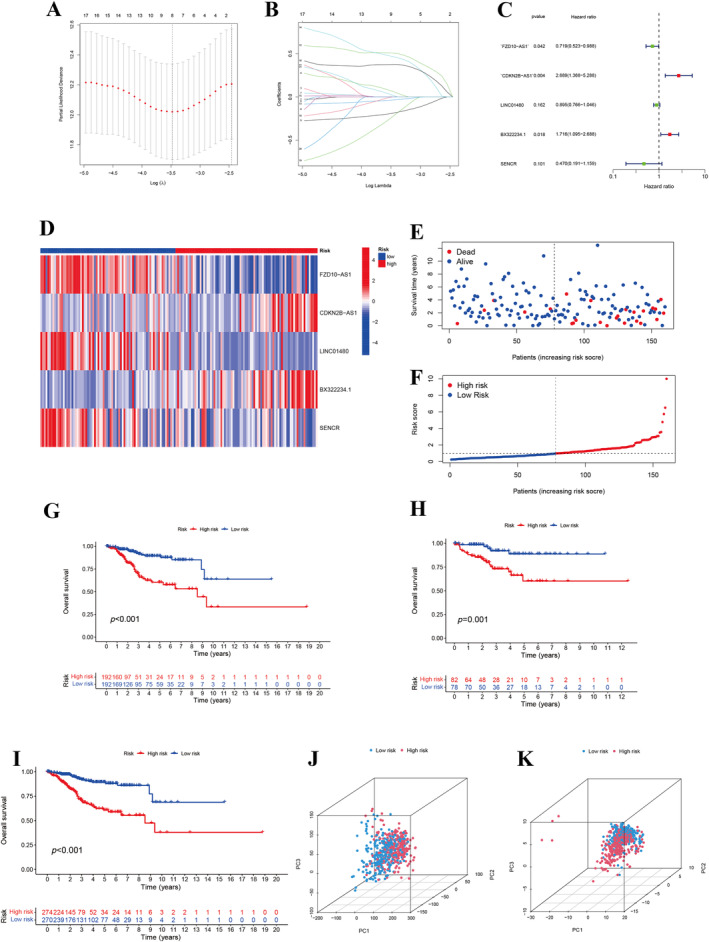
Construction of prognosis model. (A and B) LASSO Cox regression for optimal predictor selection and complexity reduction. (C) Stepwise Cox regression to establish a prognostic framework. (D) Risk heatmap in test set. (E and F) Survival status of OC patients. Kaplan–Meier (K‐M) curves between high‐risk and low‐risk groups in training set (G), test set (H), and all set (I). Principal component analysis (PCA) differentiation between training (J) and test (K) cohorts.

The K‐M survival curves revealed a markedly higher OS rate in the low‐risk group than in the high‐risk group, as confirmed by the log‐rank test (*p* < 0.001). This significant disparity underscores the prognostic value of our risk stratification. Based on risk scores, we generated the risk heatmap and survival status of two group patients. The high‐risk and low‐risk groups were clearly distinguished by PCA analysis of 21 mfclncRNAs expression (Figure 4).

### 3.3 Validation and Construction of the Prognosis Model in EC Patients

**FIGURE 5:**
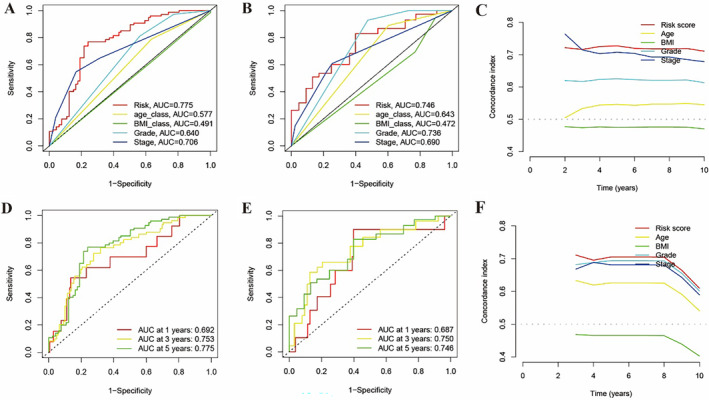
Unveiling the predictive power of the mfclncRNA signature for cancer prognosis. Demonstrating risk score superiority with 5‐year ROC curve analysis against traditional clinical markers in training (A) and test cohorts (B). ROC curve insights for predictive accuracy at 1‐, 3‐, and 5‐year intervals using the refined model in both training (D) and test (E) environments. Prognostic model precision gauged by C‐index across training (C) and testing (F) phases.

To investigate whether the risk scores can serve as an individual prognosis factor, we integrated the risk scores and corresponding clinical information to constructed mfc‐clinical prognosis model and forest plots in TCGA cohort, which showed that the risk scores work (Figure 5).

The result of ROC curves showed that AUC reached 0.692 at 1 year, 0.753 at 3 years, 0.775 at 5 years, indicating the diagnosis accuracy of prognosis model (Figure 5D,E). In addition, 5‐year ROC curves between risk score and other clinical characters were presented that the AUC of risk score reached 0.775 (Figure 5A,B), with a better diagnostic accuracy compared to other clinical features. Using a bootstrap methodology with 1000 resamples, the 5‐year C‐index for the training set was calculated to be 0.76, featuring a 95% confidence interval (CI) of 0.73–0.79 (Figure 5C). In comparison, the test set exhibited a 5‐year C‐index of 0.77, with a 95% CI of 0.74–0.80, as shown in Figure 5F. The plot of time related C‐index showed that the risk scores were more representative and sensitive than any other clinical characteristics in gene‐clinical model (Figure 5C,F).

**FIGURE 6:**
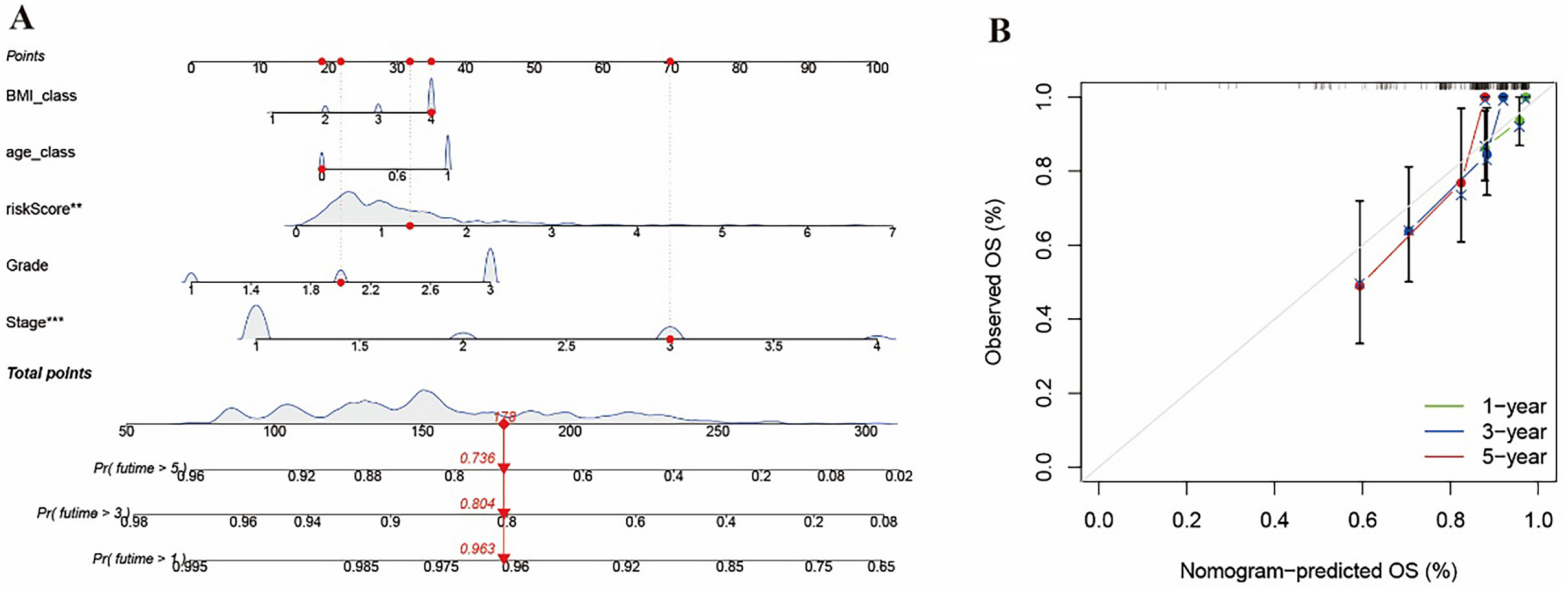
Development of a nomogram based on mfcrlncRNA signature used to predict the survival of EC patients. (A) Predictive nomograms of EC patients. (B) Corresponding calibration curves of nomograms.

Based on the prognosis model, we generated the predictive nomogram (Figure 6A). To further verify, the corresponding calibration curves predicted the prognosis of 1, 3, and 5 years of OC patients in training set (Figure 6B).

### 3.4 Gene Function Enrichment Analysis

**FIGURE 7:**
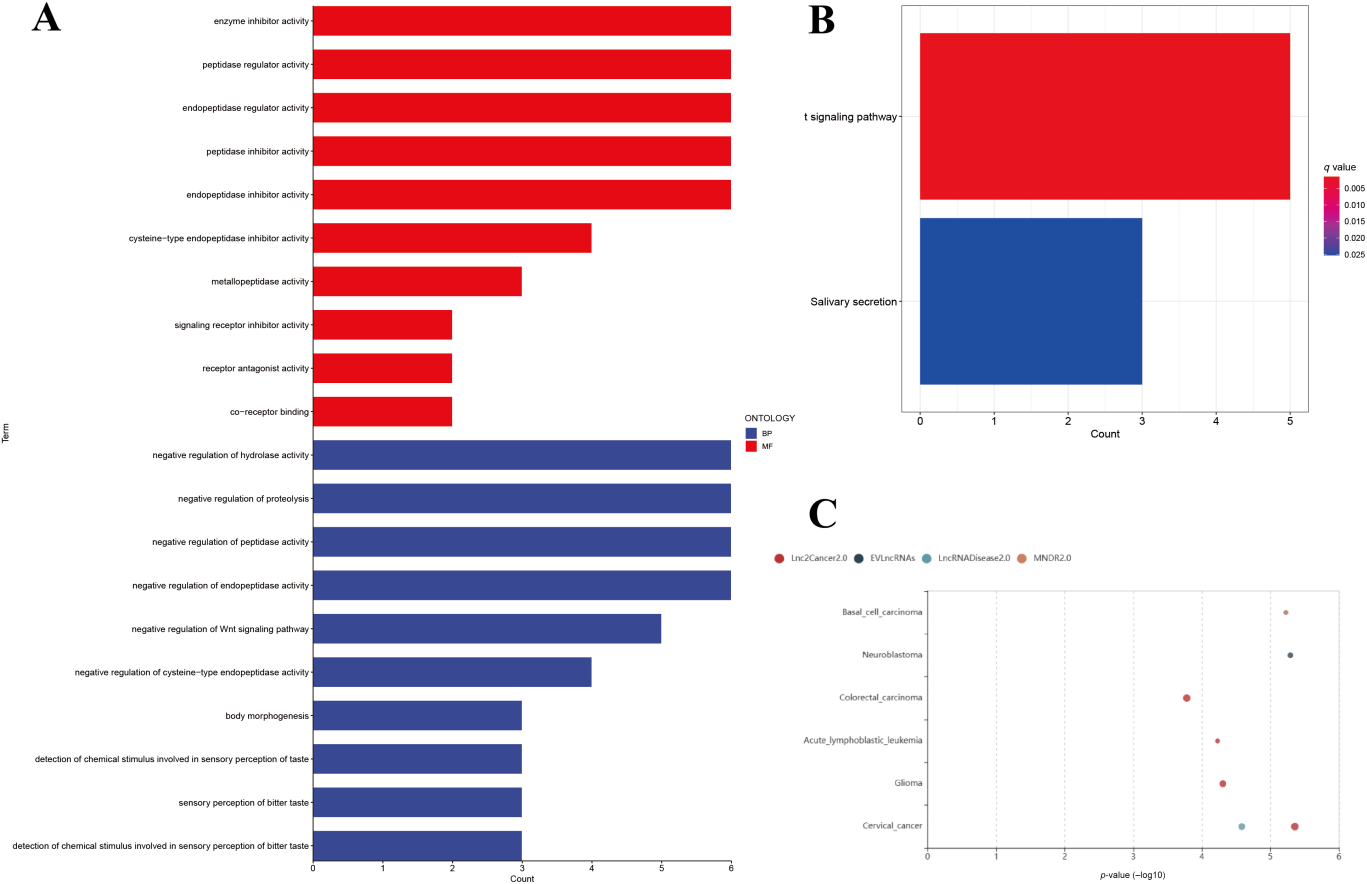
Gene function enrichment analysis of different expression signatures in high‐risk and low‐risk group. (A) GO analysis results of DESs in two risks groups. (B) KEGG pathways of DESs in two risks groups. (C) LncSEA enrichment analysis of identified mfclncRNAs.

Utilizing LncSEA, a detailed and comprehensive platform designed for the documentation and analysis of human lncRNA sets, we have been able to annotate and perform enrichment analyses on lncRNAs. The results of gene set enrichment in LncSEA present that the five identified mfclncRNAs participated in the process of nerve and mucosal tumors, including basal cell cancer, colorectal cancer, acute lymphoblastic leukemia, glioma, and cervical cancer (Figure 7C).

In an effort to decode the biofunctional roles and pathway involvements of prognostic signatures for patients in distinct risk categories, comprehensive analyses using GO and KEGG were undertaken within the expression profiles (Figure 7A). Within the domain of biological processes, the identified signatures were predominantly involved in the negative regulation of proteolysis, the Wnt signaling pathway, hydrolase activity, and peptidase activity. In terms of molecular functionality, the differentially expressed signatures (DESs) were primarily associated with roles in enzyme inhibitor expression and translational activities. This association was particularly evident in functions such as cysteine‐type endopeptidase inhibitor activity, receptor antagonist activity, and signaling receptor inhibitor activity. The enrichment in these activities underscores the DESs' critical involvement in inhibiting enzymatic actions and interfering with receptor‐mediated signaling pathways. KEGG results further confirmed that these DESs were significantly associated with the Wnt signaling pathway (Figure 7B).

### 3.5 Difference of the Tumor Mutation Burden Between High‐Risk and Low‐Risk Groups

**FIGURE 8:**
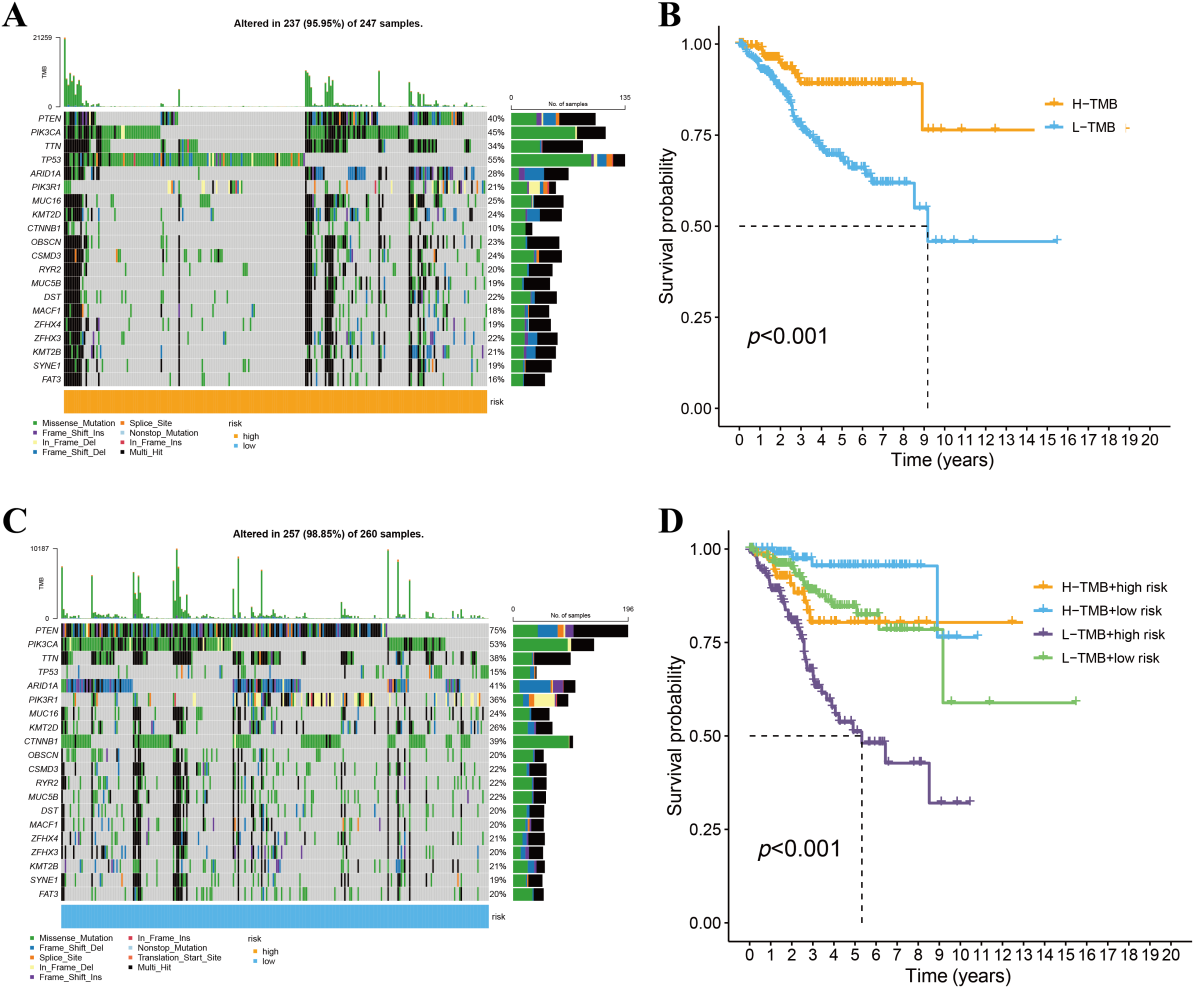
(A and C) Waterfall chart illustrating the mutation profiles of 15 genes across various risk groups. (B) Survival analysis via Kaplan–Meier curves for patients categorized into high and low TMB groups. (D) Survival curves via Kaplan–Meier analysis for EC patients, showcasing differences based on tumor mutational burden (TMB) and assigned risk scores.

We downloaded the tumor cell mutation data from the TCGA for EC patients and calculated the TMB scores. The waterfall plot showed that the high‐risk cohort differed in the composition of tumor mutation genes compared to the low‐risk cohort, and the main mutated genes in the order of mutation from high to low were TP53, PIK3CA, TTN, etc. (Figure 8A,C). The mutation rate of PTEN is notably lower in the low‐risk group compared to the high‐risk group. The samples were divided into high TMB group and low TMB group based on median TMB score, and the effect of tumor mutation load grouping on the survival rate of EC patients was investigated using OS as the outcome. The analysis revealed that patients characterized by a high TMB exhibited improved survival rates. Notably, within this cohort, individuals identified as low‐risk based on high TMB levels demonstrated the most favorable survival outcomes, suggesting a significant prognostic interplay between TMB status and risk stratification.

### 3.6 Evaluation of Immune Infiltration and Immunotherapy Effectiveness

The condition of the immune microenvironment within tumor tissues plays a crucial role in determining the prognosis of tumor patients. Through the CIBERSORT algorithm, we evaluated IS, tumor purity (TP), and estimate scores (ES) of EC patients. Our findings highlighted an increased infiltration of monocytes, macrophages M1, activated dendritic cells, and T cells follicular helper in the high‐risk group (Figure 8B). In our quest to decipher the impact of the immune microenvironment on tumor progression, we conducted a correlation analysis focusing on five specific lncRNAs. The results revealed a substantial connection between the expression patterns of these lncRNAs and the variety of immune cells present in tumor environments. This finding underscores the complex interplay between lncRNA expression and immune cell infiltration in the context of tumor biology (Figure 8C).

**FIGURE 9:**
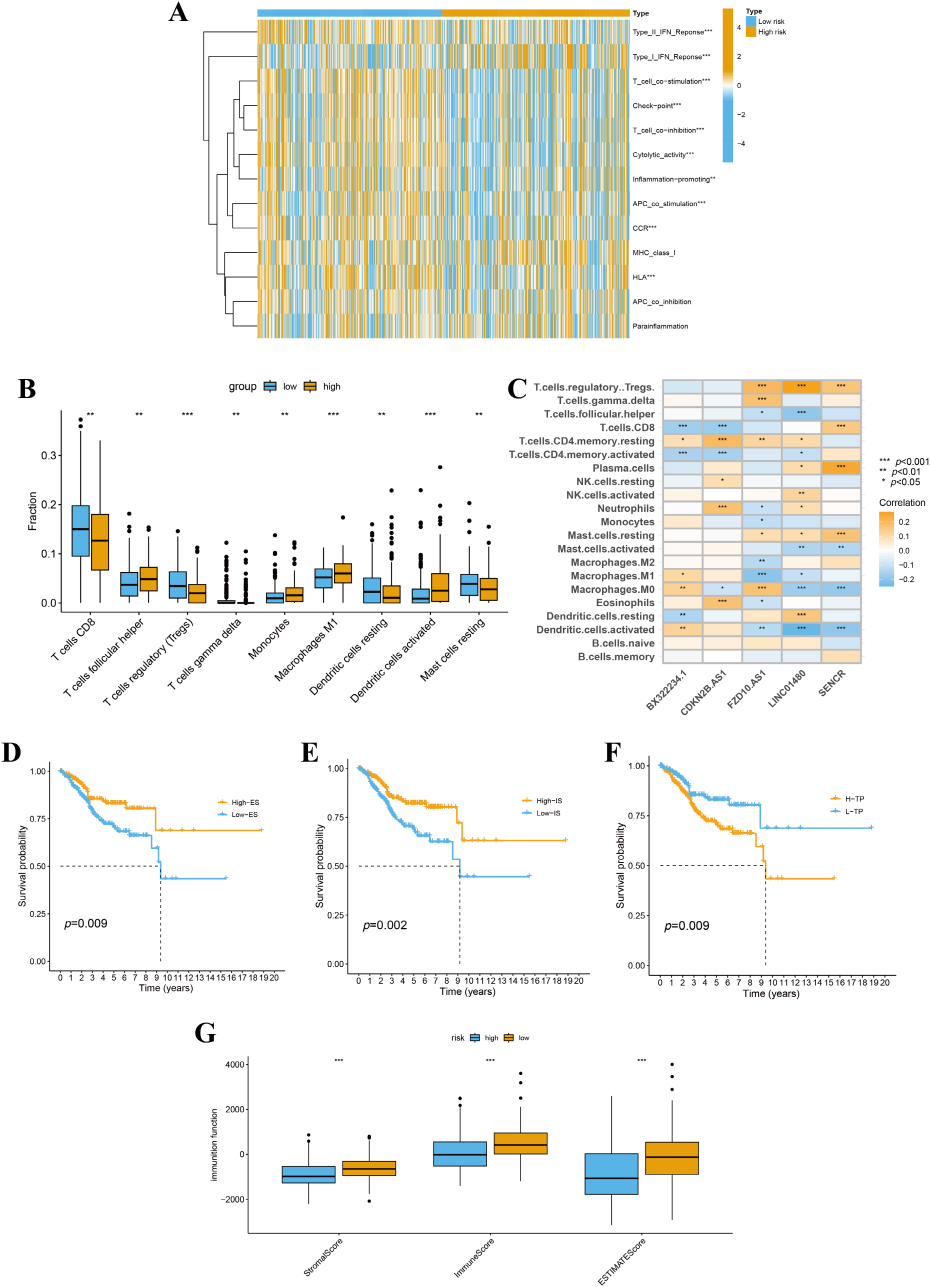
Comprehensive assessment of immune infiltration and immunotherapy efficacy. (A) Immune function comparison between high‐risk and low‐risk groups. (B) Assessment of nine immune cells' infiltration using CIBERSORT analysis in high‐risk and low‐risk groups. (C) The correlation analysis for prognostic signatures and immune cell profiles. Survival analysis of estimate score (D), immune score (E), and tumor purity (F). (G) Comparison of immune function for high‐risk and low‐risk groups.

The figure illustrates that within the high‐risk group, there is a notable decrease in Type II interferon (IFN) response, cytolytic activity, and HLA activity, contrasted by an increase in Type I IFN response (Figure 9A). Concurrently, the figure demonstrates that EC patients, identified by high ES, high IS, and low TP, exhibit enhanced survival rates and superior prognostic outcomes (Figure 9D–G).

**FIGURE 10:**
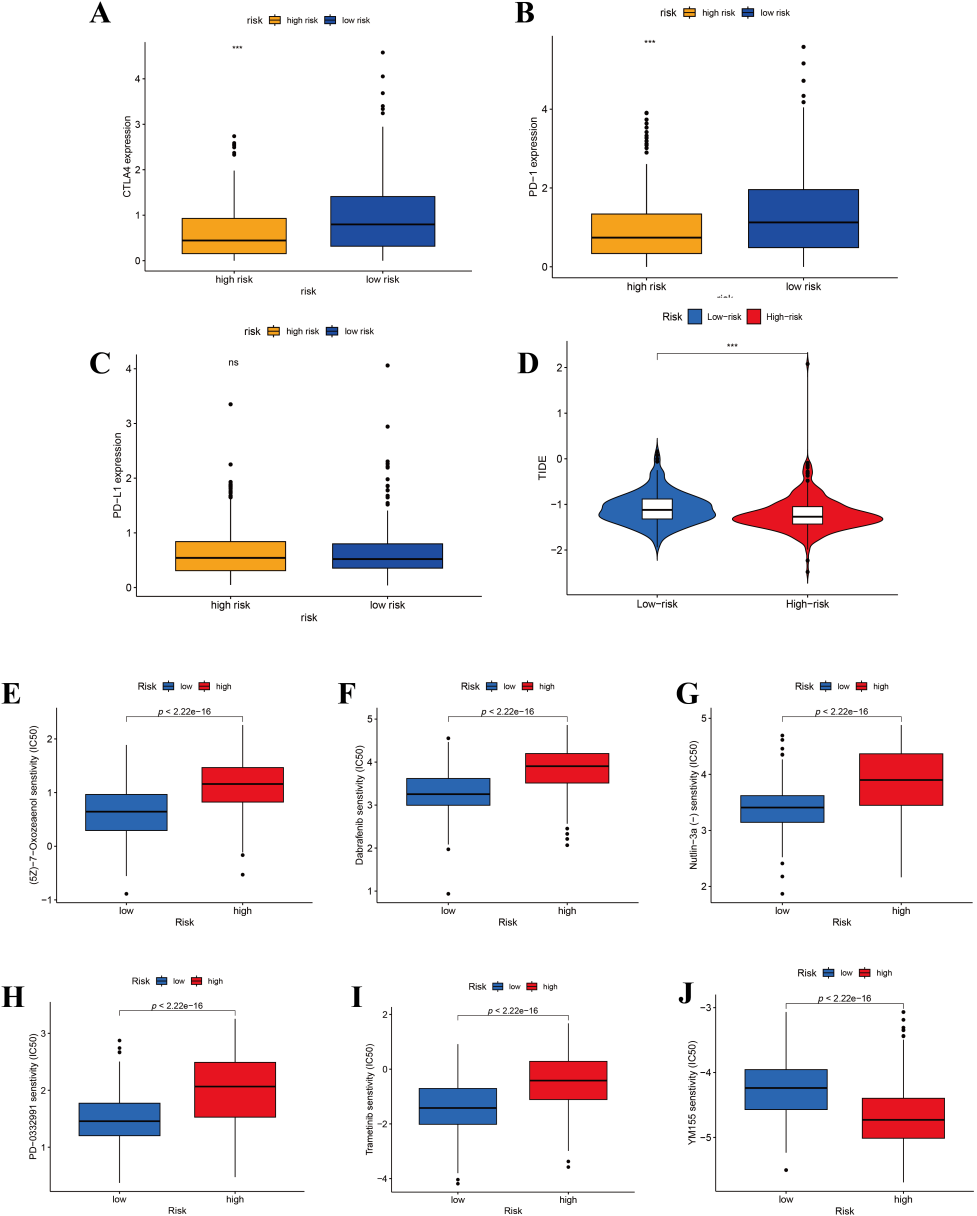
(A–C) Different expression analysis of immune checkpoint in high‐ and low‐risk group. (D) TIDE score of EC patients in two risk groups. (E–J) Drug sensitivity analysis of EC patients. ***p < 0.001.

Our evaluation of TIDE scores, calculated using differential expression signatures (DESs), revealed a distinct comparison between high‐risk and low‐risk groups (Figure 10A–D). Interestingly, individuals in the low‐risk group demonstrated elevated TIDE scores relative to those in the high‐risk group. This elevation implies an increased likelihood of immune escape in the low‐risk group, pointing to the nuanced interplay between tumor genetics and the immune environment's ability to combat cancer progression.

### 3.7 Drug Sensitivity Analysis

In order to identify potential pharmacological interventions for patients, we conducted a comparative analysis of the IC50 values for six drugs deemed effective in treatment, stratifying between high‐risk and low‐risk groups according to our established risk model (Figure 10). Within this study cohort, individuals identified as belonging to the high‐risk group exhibited increased susceptibility to a specific set of drugs, namely 5z‐7‐oxozeaenol, dabrafenib, trametinib, nutlin‐3a, and PD‐0332991 (Figure 10E). Conversely, patients assigned to the low‐risk category showed an increased responsiveness to YM155, highlighting the potential for differential drug efficacy based on risk stratification. This enhanced sensitivity in the low‐risk group points to the potential for tailored therapeutic strategies that consider individual risk profiles, especially in treatments involving YM155 (Figure 10).

## 4 Discussion

Despite advancements in surgical techniques and chemotherapy regimens for EC, the prognosis for patients often remains poor, significantly impacting patient survival. Besides, a subset of women diagnosed with EC, particularly those of reproductive age, may suffer significant psychological impacts from the loss of fertility if standard treatments involving salpingo‐oophorectomy are uniformly applied [41]. In recent years, the safety of vitrification techniques for human oocytes and embryos has been widely validated [42]. The development and maturation of these technologies have propelled advancements in assisted reproductive technologies (ART), providing more options for EC patients who wish to preserve their fertility [43]. Nevertheless, the effectiveness of fertility‐sparing treatments can be influenced by the patient's physiological characteristics and the progression of the tumor [44, 45]. Therefore, developing optimized molecular prognostic and predictive models for EC patients is crucial. These models not only facilitate the rapid identification of prognostic markers and assessment of mortality risk, thereby aiding in clinical decision‐making, but they also allow for personalized treatment plans based on the molecular characteristics of early‐stage EC [46]. This approach can evaluate the feasibility of fertility preservation techniques during cancer treatment, offering hope and support to patients wishing to maintain their fertility. In addition, increasingly evidences indicated that the cuproptosis‐, ferroptosis‐, and m6A methylation‐related lncRNAs played the potential roles in predicting the prognosis of cancers [47]. Therefore, we utilize cuproptosis‐, ferroptosis‐, and m6A methylation‐related lncRNAs to construct prognostic models for EC patients, aiming to identify biomarkers that may influence the prognosis of EC patients. These models may not only help in the rapid identification of prognostic markers and assessment of mortality risk, providing a comprehensive risk evaluation and guiding tailored treatment strategies, but also facilitate the formulation of personalized treatment plans based on the molecular characteristics of early‐stage EC. This approach can evaluate the feasibility of fertility preservation techniques during cancer treatment, may offer hope and support to patients wishing to maintain their reproductive potential.

In our investigation, we extracted co‐expressed lncRNA expression data from the TCGA cohort to identify critical signatures through a series of analytical methods: univariate Cox regression, LASSO Cox regression, and stepwise Cox regression analyses. Although we attempted to utilize machine learning methods to construct predictive models, our comparative analysis revealed that these models did not outperform the LASSO Cox regression model. Research indicates that due to the complexity of real‐world data, the Cox proportional hazards regression model provides predictive accuracy comparable to that of machine learning models while offering advantages in variable interpretability [48]. Consequently, we opted to use the LASSO Cox regression model to develop the prognostic model for EC patients. This comprehensive approach allowed us to uncover relationships between identified genes, clinical characteristics, and potential immunotherapy effects. We successfully established a prognostic model based on five multifunctional lncRNAs (mfclncRNAs)—FZD10‐AS1, CDKN2B‐AS1, LINC01480, BX322234.1, and SENCR—and calculated the model's risk scores. These signatures have been implicated in EC, reinforcing the relevance of our findings. Previous literature has highlighted the prognostic significance of these lncRNAs in various cancers. For instance, Li et al. recognized FZD10‐AS1 as a prognostic marker in colorectal cancer through the construction of a competitive endogenous RNA network [49]. This study's observation that FZD10‐AS1 is downregulated in high‐risk populations mirrors our own findings, suggesting its broader applicability across cancer types. Similarly, LINC01480's abnormal expression has been linked to the progression of multiple tumors, including its role in activating the PI3K/AKT pathway, a critical route for cell proliferation and inhibition of apoptosis [9, 50, 51, 52, 53]. This pathway's potential as a therapeutic target for EC underscores the importance of our results [51, 54]. Moreover, BX322234.1's role in regulating genomic instability and its high expression in high‐risk groups point to its significance as a biomarker for aggressive tumor behavior [3, 55]. Therefore, further investigating the biological functions related to BX322234.1 would be of significant interest and importance. The oncogenic potential of CDKN2B‐AS1 across different cancers, including its regulatory effects on miR‐378b in lung cancer, further validates the multifaceted roles of these lncRNAs in oncogenesis [56, 57]. Lastly, SENCR's involvement in vascular development and its newly discovered contribution to cisplatin resistance in non‐small cell lung cancer highlight the expansive impact of lncRNAs on cancer biology and treatment resistance [58, 59, 60]. However, the role and underlying mechanisms of SENCR in malignant tumors remain largely unexplored [61]. Our research supports the need for further in‐depth investigations into this lncRNA as a potential factor influencing cancer susceptibility.

The functional enrichment analysis delineated the pivotal roles of mfclncRNAs, predominantly associated with tumor progression and immune responses. Notably, within the GO terms, endopeptidase activity emerged as a crucial enzymatic function, facilitating protein synthesis, folding, and protection by cleaving peptide bonds in proteins. This activity plays a critical role in thwarting tumor progression by maintaining protein homeostasis [62]. Additionally, the analysis underlined biological processes mainly associated with inhibiting protease and hydrolase activities. According to KEGG pathway insights, the Wnt signaling pathway stands out for its integral role in both embryonic development and the emergence of tumors [63, 64].

To corroborate the validity of the risk score within our prognostic model, K‐M curves were generated, revealing significant survival discrepancies between the high‐risk and low‐risk groups. Further analytical rigor was provided by plotting the C‐index and its time‐dependent variant, both of which underscored the prognostic model's risk scores as a potent individual prognostic factor, offering distinct advantages over traditional clinical characteristics. To extend the validation process, a testing set was employed to craft a predictive nomogram alongside corresponding calibration curves. These tools not only affirmed the risk score's predictive capacity for the prognosis of EC patients but also facilitated a deeper investigation into the prognostic model's efficacy.

In exploring the gene mutation profiles across different risk groups, we found that PTEN has a lower mutation rate in the low‐risk group with better prognosis. Conversely, ARID1A and TP53 exhibit higher mutation rates in the high‐risk group with poorer prognosis. This finding corroborates the results reported by Gullo et al. [65]. In our endeavor to elucidate the functional impact of immune cell infiltration on EC, we delved into the association between prognostic lncRNA signatures and the extent of immune cell penetration within the tumor microenvironment. Our findings reveal that EC patients characterized by elevated ES, enhanced IS, and diminished TP are correlated with improved survival rates and more favorable prognostic indicators. The tumor microenvironment of EC is a complex network composed of a variety of cell types, including endothelial cells, fibroblasts, myofibroblasts, alongside key immune and inflammatory cells. Consistent with prior research, our analysis substantiates that the presence of tumor‐infiltrating immune cells plays a significant role in determining the prognosis of EC, underscoring the critical interplay between immune infiltration and cancer progression [66, 67]. Our findings excitingly reveal that, through risk stratification, patients categorized as high risk exhibit a pronounced infiltration of CD8^+^ cells. As crucial entities for eradicating cancer cells, CD8^+^ cytotoxic T lymphocytes are instrumental in both the development and assessment of immunotherapeutic strategies for combating cancer. This observation underscores the vital role these cells play in influencing patient outcomes. Conversely, individuals identified as low risk display more robust immune responses, correlating with superior prognostic results. Further examination of the relationship between risk scores and immune functionalities uncovered that high‐risk patients exhibit reduced activities in Type II IFN response, T‐cell co‐stimulation, T‐cell co‐inhibition, cytolytic activity, and antigen‐presenting cell (APC) co‐stimulation, whereas Type I IFN response remains notably active. The synergistic modulation of Type I and Type II responses plays a critical role in activating pro‐inflammatory pathways, while also initiating inhibitory feedback mechanisms that challenge cancer control efforts [68]. Particularly, the Type I IFN response, known for regulating both self‐immunity and antitumor activities, may under certain conditions impair self‐immune reactions or activate feedback loops that limit effective immune responses [69, 70]. The downregulation of Type II IFN response suggests a potential mechanism for immune evasion, leading to less favorable outcomes for high‐risk EC patients [8]. Moreover, our research aligns with studies on various cancer types, demonstrating that TMB is a predictive marker for the efficacy of immune checkpoint inhibitors [71, 72]. Significantly, EC patients with elevated TMB levels were associated with better prognoses, with the low‐risk group displaying higher TMB values. Analysis of TIDE scores between the high‐risk and low‐risk groups indicated a greater likelihood of immune escape phenomena in the former. Collectively, these findings highlight that the low‐risk group benefits from increased antitumor immune activities, offering insight into the prognostic significance of the identified features.

In addition, we evaluated the effects of immunotherapy. Cancer immunotherapy has demonstrated efficacy in treating various malignant tumors by either activating systemic immune responses or restoring immune defects caused by tumors [73]. Several case reports have shown the effectiveness of immune checkpoint inhibitors targeting receptors such as PD‐1, PD‐L1, and CTLA4 in late‐stage or metastatic EC patients [74, 75]. Based on pharmacological sensitivity analysis, our findings indicate that 5z‐7‐oxozeaenol, dabrafenib, trametinib, nutlin‐3a, and PD‐0332991 exhibit heightened sensitivity in patients categorized as high‐risk, whereas YM155 demonstrates increased sensitivity in those classified under the risk group. The variance in drug sensitivity is correlated with the molecular characteristics and genetic mutations specific to these patient groups. The loss of PTEN activates the PI3K/AKT signaling pathway, augmenting the dependency of cells on survivin. Under these circumstances, the application of the survivin inhibitor YM155 can more effectively suppress the proliferation of tumor cells [76]. Within cells harboring TP53 mutations that modulate the p53 pathway, the TAK1 inhibitor 5Z‐7‐oxozeaenol can restore apoptotic functions in a subset of cells by inhibiting the NF‐κB signaling pathway, which bolsters the antitumor potency of chemotherapy agents [77]. Loss of PTEN boosts PI3K/AKT signaling, potentially leading to resistance against BRAF and MEK inhibitors like dabrafenib and trametinib [78]. PD‐0332991, a CDK4/6 inhibitor, enhances therapeutic outcomes by disrupting cell cycle regulation in tumor cells, particularly those lacking TP53‐mediated apoptosis and repair [79]. Recent clinical trials of trametinib and dabrafenib for EC patients showed partial responses in three patients [80, 81]. Clinical investigations suggest that PD‐0332991, a selective cyclin‐dependent kinase 4/6 inhibitor, has shown promise in treating EC, suggesting its potential applicability in a broader range of malignancies [82]. In summary, these pharmacological discoveries enable us to integrate prognostic models to assess risk factors in diverse patient populations, thereby facilitating a more tailored and personalized approach to treatment. However, further clinical trials are essential to refine these strategies.

Our study demonstrates notable strengths, including the use of gene expression profiles and clinical data from two public platforms, enhancing our prognosis model's reliability and validity. The model's risk scores were validated internally, reinforcing their role as potential prognostic indicators. Despite unequal baselines between training and test sets, both validations supported the risk scores' predictive value. Additionally, we analyzed the biofunction and immune response of identified signatures, offering insights into potential EC therapies. However, our study is not without limitations. A notable challenge was the incomplete clinical data from TCGA for EC, particularly the absence of TNM staging information. This gap underscores the need for comprehensive clinical data to strengthen future research. Moreover, to solidify the prognostic utility of the identified mfclncRNAs in EC, further validation through corresponding cellular and animal experiments is essential. These steps are crucial for translating our findings into practical therapeutic strategies.

## 5 Conclusion

In our investigation, we pinpointed five pivotal lncRNAs in EC via the development of a prognostic model. These lncRNAs' associated risk scores were confirmed as effective predictors of prognostic outcomes in EC patients. Subsequent evaluations incorporating clinical features, functional enrichment studies, and assessments of immunotherapy responses underscored the significant promise of these lncRNA signatures as innovative therapeutic and diagnostic markers for EC.

## Author Contributions

**Yongkang Qian:** conceptualization, methodology, software, formal analysis, resources, writing – original draft. **Hualing Chen:** software, data curation, validation, project administration. **Pengcheng Miao:** methodology, software, validation. **Rongji Ma:** investigation, software, validation. **Beier Lu:** methodology, visualization. **Chenhua Hu:** project administration, software. **Ru Fan:** project administration, supervision. **Biyun Xu:** conceptualization, writing – review and editing. **Bingwei Chen:** conceptualization, supervision, writing – review and editing.

## Conflicts of Interest

The authors declare no conflicts of interest.

## Supporting information


**Figure S1.** Correlation analysis diagram of risk score and drug sensitivity IC50.

**Table S1.** C‐index of mfclncRNA prognostic model.

**Table S2.** GO analysis in high‐risk and low‐risk groups.

## Data Availability

All data utilized in this research are accessible through the TCGA database, available at (https://portal.gdc.cancer.gov/).
