# Supplementary figures and images for: Integrated Identification and Immunotherapy Response Analysis of the Prognostic Signature Associated With m6A, Cuproptosis‐Related, Ferroptosis‐Related lncRNA in Endometrial Cancer
Source: Cancer Rep (Hoboken). 2024 Sep 26;7(9):e70009. doi: 10.1002/cnr2.70009 (PMC11425647; doi:10.1002/cnr2.70009)

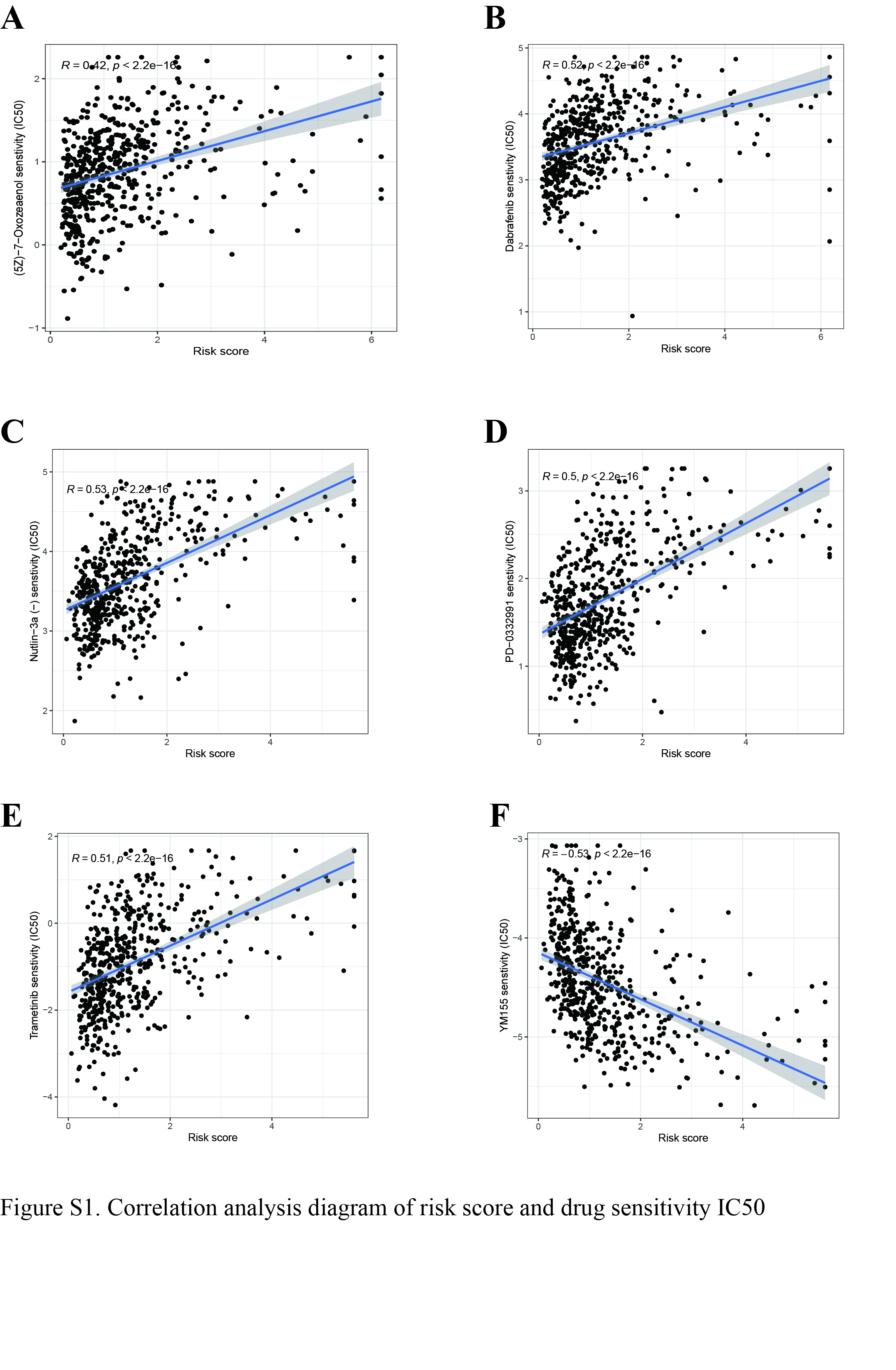

Supplement: Supplementary file 1 — Figure S1. Correlation analysis diagram of risk score and drug sensitivity IC50. [file CNR2-7-e70009-s002.jpg]
