# Supplementary material for: Integrated Identification and Immunotherapy Response Analysis of the Prognostic Signature Associated With m6A, Cuproptosis‐Related, Ferroptosis‐Related lncRNA in Endometrial Cancer
Source: Cancer Rep (Hoboken). 2024 Sep 26;7(9):e70009. doi: 10.1002/cnr2.70009 (PMC11425647; doi:10.1002/cnr2.70009)
Supplement: Supplementary file 2 — Table S1. C‐index of mfclncRNA prognostic model. [file CNR2-7-e70009-s001.pdf]

Table S1. C index of mfclncRNA prognostic model.

Data	C index	95% lower confidence interval	95% higher confidence interval
Train	0.76	0.727	0.793
Test	0.77	0.737	0.803
