# Supplementary material for: Integrated Identification and Immunotherapy Response Analysis of the Prognostic Signature Associated With m6A, Cuproptosis‐Related, Ferroptosis‐Related lncRNA in Endometrial Cancer
Source: Cancer Rep (Hoboken). 2024 Sep 26;7(9):e70009. doi: 10.1002/cnr2.70009 (PMC11425647; doi:10.1002/cnr2.70009)
Supplement: Supplementary file 3 — Table S2. GO analysis in high‐risk and low‐risk groups. [file CNR2-7-e70009-s003.pdf]

Table S2. GO analysis in different risk groups.

ONTOLOGY	ID	Description	GeneRatio	BgRatio	pvalue	p.adjust	qvalue	geneID	Count
BP	GO:0010951	negative regulation of endopeptidase activity	6/34	252/18903	5.20E-06	0.002618	0.002011	CST4/DPEP1/SERPINA6/CST2/CST1/SERPINA4	6
BP	GO:0010466	negative regulation of peptidase activity	6/34	263/18903	6.64E-06	0.002618	0.002011	CST4/DPEP1/SERPINA6/CST2/CST1/SERPINA4	6
BP	GO:0030178	negative regulation of Wnt signaling pathway	5/34	174/18903	1.40E-05	0.003673	0.002821	WIF1/NOTUM/DKK4/DKK1/NKD1	5
BP	GO:2000117	negative regulation of cysteine-type endopeptidase activity	4/34	91/18903	2.09E-05	0.004111	0.003158	CST4/DPEP1/CST2/CST1	4
BP	GO:0045861	negative regulation of proteolysis	6/34	352/18903	3.46E-05	0.005203	0.003996	CST4/DPEP1/SERPINA6/CST2/CST1/SERPINA4	6
BP	GO:0001580	detection of chemical stimulus involved in sensory perception of bitter taste	3/34	37/18903	3.96E-05	0.005203	0.003996	CST4/CST2/CST1	3
BP	GO:0051346	negative regulation of hydrolase activity	6/34	373/18903	4.78E-05	0.005379	0.004132	CST4/DPEP1/SERPINA6/CST2/CST1/SERPINA4	6
BP	GO:0050913	sensory perception of bitter taste	3/34	42/18903	5.82E-05	0.005469	0.004201	CST4/CST2/CST1	3
BP	GO:0050912	detection of chemical stimulus involved in sensory perception of taste	3/34	43/18903	6.25E-05	0.005469	0.004201	CST4/CST2/CST1	3
BP	GO:0010171	body morphogenesis	3/34	48/18903	8.70E-05	0.006856	0.005266	GREM2/DKK1/IHH	3
BP	GO:0052548	regulation of endopeptidase activity	6/34	428/18903	0.000102	0.00732	0.005623	CST4/DPEP1/SERPINA6/CST2/CST1/SERPINA4	6
BP	GO:0090090	negative regulation of canonical Wnt signaling pathway	4/34	141/18903	0.000116	0.007592	0.005831	NOTUM/DKK4/DKK1/NKD1	4
BP	GO:0052547	regulation of peptidase activity	6/34	459/18903	0.00015	0.009078	0.006973	CST4/DPEP1/SERPINA6/CST2/CST1/SERPINA4	6
BP	GO:0050909	sensory perception of taste	3/34	68/18903	0.000246	0.013845	0.010634	CST4/CST2/CST1	3
BP	GO:0048070	regulation of developmental pigmentation	2/34	14/18903	0.000282	0.013884	0.010664	EDN3/IHH	2
BP	GO:0120305	regulation of pigmentation	2/34	14/18903	0.000282	0.013884	0.010664	EDN3/IHH	2
BP	GO:0030111	regulation of Wnt signaling pathway	5/34	336/18903	0.000314	0.014224	0.010926	WIF1/NOTUM/DKK4/DKK1/NKD1	5
BP	GO:2000052	positive regulation of non-canonical Wnt signaling pathway	2/34	15/18903	0.000325	0.014224	0.010926	DKK1/NKD1	2
BP	GO:2000095	regulation of Wnt signaling pathway, planar cell polarity pathway	2/34	16/18903	0.000371	0.015383	0.011816	DKK1/NKD1	2
BP	GO:0061053	somite development	3/34	86/18903	0.000491	0.019358	0.014869	DKK1/IHH/NKD1	3
BP	GO:2000116	regulation of cysteine-type endopeptidase activity	4/34	235/18903	0.000806	0.030229	0.023219	CST4/DPEP1/CST2/CST1	4
BP	GO:2000050	regulation of non-canonical Wnt signaling pathway	2/34	26/18903	0.000993	0.035578	0.027328	DKK1/NKD1	2
BP	GO:0060828	regulation of canonical Wnt signaling pathway	4/34	260/18903	0.001172	0.040115	0.030812	NOTUM/DKK4/DKK1/NKD1	4
BP	GO:0016055	Wnt signaling pathway	5/34	456/18903	0.001248	0.040115	0.030812	WIF1/NOTUM/DKK4/DKK1/NKD1	5
BP	GO:0198738	cell-cell signaling by wnt	5/34	458/18903	0.001273	0.040115	0.030812	WIF1/NOTUM/DKK4/DKK1/NKD1	5
BP	GO:0060323	head morphogenesis	2/34	36/18903	0.001904	0.057703	0.044322	DKK1/IHH	2
MF	GO:0004866	endopeptidase inhibitor activity	6/33	180/18432	7.10E-07	4.36E-05	3.16E-05	CST4/DPEP1/SERPINA6/CST2/CST1/SERPINA4	6
MF	GO:0030414	peptidase inhibitor activity	6/33	187/18432	8.88E-07	4.36E-05	3.16E-05	CST4/DPEP1/SERPINA6/CST2/CST1/SERPINA4	6
MF	GO:0061135	endopeptidase regulator activity	6/33	194/18432	1.10E-06	4.36E-05	3.16E-05	CST4/DPEP1/SERPINA6/CST2/CST1/SERPINA4	6
MF	GO:0004869	cysteine-type endopeptidase inhibitor activity	4/33	56/18432	2.93E-06	7.39E-05	5.36E-05	CST4/DPEP1/CST2/CST1	4
MF	GO:0061134	peptidase regulator activity	6/33	232/18432	3.11E-06	7.39E-05	5.36E-05	CST4/DPEP1/SERPINA6/CST2/CST1/SERPINA4	6
MF	GO:0004857	enzyme inhibitor activity	6/33	395/18432	6.33E-05	0.001255	0.00091	CST4/DPEP1/SERPINA6/CST2/CST1/SERPINA4	6
MF	GO:0039706	co-receptor binding	2/33	13/18432	0.000239	0.004071	0.002953	DKK4/DKK1	2
MF	GO:0048019	receptor antagonist activity	2/33	32/18432	0.001491	0.022176	0.016085	DKK4/DKK1	2
MF	GO:0030547	signaling receptor inhibitor activity	2/33	43/18432	0.002681	0.035448	0.025712	DKK4/DKK1	2
MF	GO:0008237	metallopeptidase activity	3/33	184/18432	0.004284	0.050984	0.036981	KLK7/DPEP1/ADAMTS19	3
